# Effects of a general practice guided web-based weight reduction program - results of a cluster-randomized controlled trial

**DOI:** 10.1186/1471-2296-14-76

**Published:** 2013-06-07

**Authors:** Michael Mehring, Max Haag, Klaus Linde, Stefan Wagenpfeil, Florian Frensch, Jasper Blome, Antonius Schneider

**Affiliations:** 1 Institute of General Practice, Technische Universität München, Orleansstr. 47, Munich 81667, Germany; 2 Institute for Medical Biometry, Epidemiology und Medical Informatics (IMBEI), Universitätsklinikum des Saarlandes, Homburg/Saar, Germany; 3 HausMed eHealth Services GmbH, Schlesische Str. 29/30 L, Berlin, 10997, Germany

**Keywords:** Overweight, Obesity, Weight loss, Web-based, Randomized controlled trial

## Abstract

**Background:**

Preliminary findings suggest that web-based interventions may be effective in achieving significant weight loss and weight loss maintenance. To date only few findings within primary care patients and especially the involvement of general practitioners are available. The aim of this trial was to examine the short-term effectiveness of a web-based coaching program in combination with an accompanied telephone counselling regarding weight reduction in a primary care setting.

**Methods:**

The study was a cluster-randomized trial with an observation period of 12 weeks. Individuals recruited by general practitioners randomized to the intervention group participated in a web-based coaching program based on education, motivation, exercise guidance, daily SMS reminding, weekly feedback through internet and active monitoring by general practitioners. Participants in the control group received usual care and advice from their practitioner without the web-based coaching program. The main outcome was weight change between admission and after 12 weeks.

**Results:**

186 participants (109 intervention group, 77 control group) were recruited into study. For 76 participants from the intervention group and 72 participants from the control group weight measurements were available both at baseline and 12 weeks. Weight decreased on average by 4.2 kg in the intervention group and 1.7 kg in the control group (mean group difference 2.5 kg; 95%CI 1,1; 3,8; p < 0.001). Reductions for waist circumference and BMI were also significantly larger within intervention.

**Conclusion:**

Findings of the present trial suggest that the tested web-based coaching program for weight loss is effective in short-term. Further RCT’s are desirable in order to confirm present findings in larger populations and to investigate long-term outcomes.

**Trial registration:**

German Register for Clinical Trials: DRKS00003067

## Background

Obesity is a growing problem, with around 1 billion people worldwide overweight and more than 300 million people obese [1]. This increase of obesity is associated with increasing incidence of major health problems such as diabetes, ischaemic heart disease and some cancers. Weight loss of 5 to 10% is associated with significant risk reduction for diabetes and cardiovascular diseases [2,3], reduction of musculoskeletal pain [4] and its associated disability [5] and increase in quality of life [6]. Many different kinds of strategies have been implemented to foster weight reduction in effective ways. Some commercial programs with evidence for effectiveness [7,8] have a particularly relevant role on the health market. These programs, however, are based on personal participation in mostly weekly in-site visits or group meetings; and it was shown that time and travel demands are associated with attrition rates of around 20% [9,10].

Web-based programs have some advantages for obese individuals who prefer to lose weight without having to participate in regular meetings. The evidence of a variety of web-based interventions was summarized in a review by Manzoni et al. [11]. However, they pointed out that comparison of a web-based intervention with a real control group in terms of usual care was missing. Another critical point is that web-based weight reduction programs with free access might bear some risk of harm if the suitability of the individual patient is not assessed by a physician prior to the intervention, particularly with respect to diabetes or eating disorders.

Therefore a web-based program was developed, which combines an individually tailored strategy for weight reduction with automated advice and feedback elements based on cognitive behavioural therapy, in addition to monitoring via internet and telephone counselling in general practice. Such a tool would facilitate the management of patients as they receive support from their general practitioner (GP) during the weight reduction process guided by the web-based program. The aim of the study was to examine the short-term effectiveness of a web-based coaching program in combination with an accompanied telephone counselling regarding weight reduction in a primary care setting.

## Methods

### Design

The study was designed as a two-armed cluster-randomized controlled trial. At the beginning of the study, around 2.000 Bavarian general practitioners (GPs) received a fax by the Bavarian Association of General Practitioners with information about the research project. All interested GPs were sequentially registered for randomization. After giving written consent, the participating practices were randomized either to the interventional or the control arm. The sequence of randomization (allocation 1:1) was provided by a methodologist, who did not participate in the execution of the study, via the program Randomizer (http://www.randomizer.org). Randomization was concealed by using sequentially numbered, opaque sealed envelopes hold by the study coordinator. Before starting recruitment of patients, physicians and practice nurses received detailed instructions by the research team on the study process (both intervention and control group) and on the coaching program (only intervention group).

Physicians assigned to the control arm were asked to change nothing in their usual way of counselling and to treat participants in the same manner as if they would have been non-participants. There was no structured documentation of the care provided.

The patients recruited by intervention practices received free access to the web-based coaching program. The patients of practices participating in the control arm were advised by the GPs in their individual way of usual care to reduce weight.

The study was approved by the Medical Ethics Committee of the Technical University of Munich on 19 April 2011 and was in accordance with ethical standards for human experimentation established by the Declaration of Helsinki. All participants gave written informed consent. A data and safety monitoring board was established before the beginning of the study. The study was registered on the German Register for Clinical Trials (http://apps.who.int/trialsearch/; registration number DRKS00003067).

### Participants and procedures

Participating physicians were general practitioners in Bavaria, Germany. The GPs were requested to recruit overweight individuals of whom a weight reduction was recommendable. Individuals with a BMI ≥ 25 were potentially eligible. Exclusion criteria were age younger than 18 year, insufficient German language skills, and lack of internet access. Further exclusion criteria were BMI < 25, Type-1-diabetes, hypothyroidism, pregnancy, breast feeding, addiction to drugs or alcohol, consuming and immune deficit disorders, heavy mental illnesses, osteoporosis, renal insufficiency, heart failure, coronary heart disease, eating disorders, cirrhosis of the liver, acute infections, other heavy metabolic illnesses (e.g. gouts).

After decision of the GP that the participation of a patient was recommendable for enrolment, an information form was given and discussed and a participation form had to be signed. At the same time the baseline data acquisition took place. All participants were asked to fill in a standardized questionnaire together with the GP. Baseline data of weight and waist circumference were measured in general practice; eating behaviour and physical activity were documented at the same time. Participants of the intervention group received a free web-code. The physician filled in a form together with the patient with detailed information about diabetes mellitus or hypertension (if necessary), dietary advices (like low cholesterol diet) and suggested physical activity. This form and the web-code are used by the patient for specification during the registration process of the internet program.

Physicians of both groups were requested to document the anthropometric measures from the participants with their existing practice equipment. The physicians were advised to measure the weight in underwear without shoes and to measure the waist circumference corresponding to the standardised definition (measured with a tape around the abdomen located marginally above the upper hip bone). A follow-up investigation with the same measurements and documentation was performed in the general practices twelve weeks after inclusion.

Physicians in the intervention group received € 50.- per participant for time and effort. Physicians in the control group received € 25.-. Participants in the intervention group received free access to the HausMed weight reduction program which usually costs € 79.-. Participants in the control group received € 10.- as an incentive to come into practice for follow-up investigation after twelve weeks. Safety monitoring in the intervention arm was guaranteed by a check of the health status four weeks after beginning of the study.

### Intervention

Anamnestic and health data were documented in a structured form including information about co-morbidities and physical activity advises from the GP. The patient received a copy of this form in order to use the health data and advises for inscribing via Internet into the coaching program (HausMed eHealth Services GmbH, Berlin; Germany; http://www.hausmed.de; indexed in Internet Archive) at home. A specific website was installed for the participants to allow a login without charge. After completion of a pre-assessment, the program generated an individual coaching based on the recommendations of the physicians, the physical characteristics and the everyday behaviour of the participants.

The coaching program is based on principals of the cognitive behavioural therapy – e.g. education, realistic goal-setting and individual resources – and in particular on the behavioural change theory targeted to reduce and maintain weight by using inexpensive Internet and mobile technologies in combination with existing health care resources of GPs. The content of the coaching program aims at achieving a lasting change of behaviour patterns with the help of individualized education, motivation, exercise guidance, daily SMS reminders, self-monitoring via Internet and, finally, through an active monitoring and approximately 3 telephone calls during the 12 weeks by the GPs or their respective staff.

The framework of the program is based on the idea by Prof. Dr. Pudel, Institute for Nutrition and Psychology Research Department at the University of Göttingen [12,13]. A weight loss plan including individual energy requirements supports each patient during the 12 weeks and assures an adequate nutrition including nutrients such as proteins, carbons and fat. The given dietary recommendations are based on current nutritional guidelines (German Nutrition Society - DGE). Additionally, the program was conceptualised for the nutritional needs of diabetics and hypertensive patients.

The coaching program is subdivided into 12 different constitutive modules. Each module is performed for one week and contains a particular exercise, which is supported by a corresponding daily SMS reminder. The goals of the particular exercises are:

• to introduce a structured diet plan

• to prevent fear for changing eating habits

• to enhance physical activity in regard to the individual condition

• to achieve a flexible control of eating behaviour

• to learn a conscious individualized food and fluid intake

• to motivate for physical activity

• to consume five portions of fruits and vegetables a day

• to understand the effect of insulin and to control blood sugar

• to understand the effect of salt respective hypertension

• to prevent failures and develop coping strategies

• to achieve a conscious handling with fatty acids

• to relief active stress

• to objective realistic goals and to frame sub-goals

• to summarize achieved goals and to motivate to continue

The reminder has an adapted content to obtain the motivation and to impart daily tips. The coaching program also offers a variety of printing material (diet plans, recipes, questionnaires, information, self-agreements etc.) which is connected to the respective exercise and includes interactive buttons, video clips and learning progress quizzes to examine the learning success. At the end of each week participants are asked to give feedback via the internet concerning their condition and level of motivation and whether or not they did their weekly task. Diary entries for weight and waist circumferences are offered as well. Participants can also communicate among themselves through a forum or ask a HausMed team member should they have any questions. The active monitoring (or rather supervising) of the entire twelve-week coaching course is carried out through a separate login account in a particularly secured physician area (weight, waist circumference, motivation, condition and status of the module exercise). In addition to that, three specified telephone calls from the GP or a qualified practice nurse (week 1, 5 and 12) are implemented to primarily motivate and support the participants. If either a participant’s motivation or condition declines notably within the weekly feedback at any point during the coaching period or the module exercise is not completed, additional counselling from the GP or practice nurse is made over the telephone. There is no limitation to the frequency of website use but participants were given a goal of using the website at least once a week and GPs are advised to log in into the program twice a week. This trial was the first evaluation of this intervention.

### Outcome measures

The primary outcome measure was weight loss in kg twelve weeks after inclusion into the study. Weight was measured at baseline and after 12 weeks. Secondary outcome measures were difference in waist circumference, BMI, eating behaviour and physical activity. The waist circumference was measured at baseline and after 12 weeks. The evaluation of the self estimated eating behaviour (range from 1–5), conscious eating pattern (range from 0–2), frequent cooking/ cooked meals (range from 0–2) and physical activity (range from 1–4) was conducted at baseline and after 12 weeks. A higher number on the scale refers to a more even or more conscious eating behaviour, more frequent cooking/ cooked meals or more frequent physical activity.

### Statistics analysis

Sample size calculation was performed with G * Power 3 correcting for the cluster design (intra-cluster correlation coefficient = 0.05, average cluster size = 3) for two-sided testing (α = 5% and a power of 80%). Using these assumptions the calculated total sample size for primary outcome weight loss was 142 participants. Taking expected attrition into account we aimed at recruiting a total of 180 participants in about 60 general practices.

Baseline data are presented descriptively. Group differences were calculated for all participants whose weight was available at baseline and follow-up (completer collective). Sensitivity analysis was performed by an intent-to-treat analysis assuming that participants with missing values had no weight change at all. The strongly variable cluster size caused major numerical problems in the linear mixed model analysis. As it was not possible to adjust for intra cluster correlations properly and because of the high number of practices with only one patient (12 practices), it was decided to perform the main analysis using Student’s t-test without accounting for the clusters. For the outcomes weight loss, changes in BMI and waist circumference we also performed secondary analyses based on generalized estimating equations with adjustment for baseline values, age, diets and occupational status. These analyses were performed as sensitivity analyses and take account of practices as patient clusters. All analyses were performed using SPSS version 19.0 (Inc., Chicago, IL).

## Results

**Figure 1:**
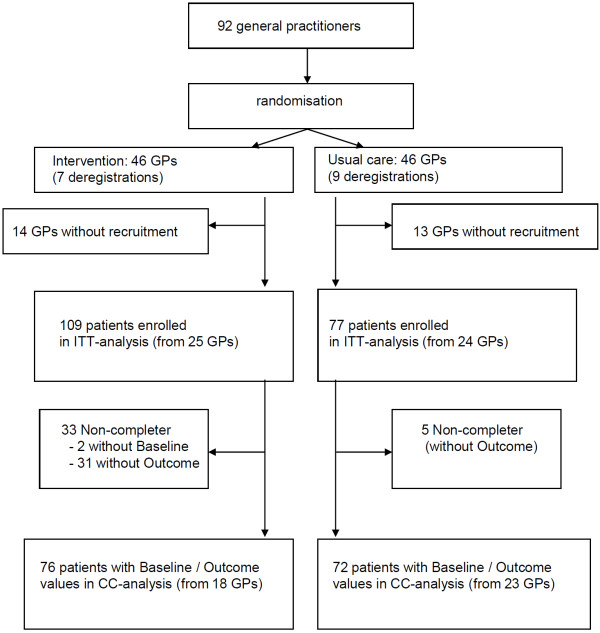
Participant flow of the study (GP = general practitioner; ITT = intention-to-treat; CC-Analysis = complete-case).

Originally 92 practices were interested to participate and were randomised. 16 practices withdrew early after randomization (7 GPs from the intervention and 9 GPs from the control group), 27 practices (14 GPs from the intervention and 13 GPs from the control group) did not recruit any participant for the study (Figure 1). Altogether, 186 patients were recruited. 128 (68.8%) participants were female, the average age was 47.8, average weight was 96.6 kg and average BMI was 33.7 kg/m^2^. 31 participants in the intervention group did not show up for the measurement at 12 weeks and 2 participants of the intervention group had missing baseline values. In the usual care group 5 participants had missing values at 12 weeks. There were no significant differences with respect to gender, weight, BMI or waist circumference at baseline between completers and non-completers.

For 148 participants (76 from the intervention and 72 from the control group) weight measurements were available both at baseline and 12 weeks (completer). Intervention and control group were similar regarding gender, baseline weight, waist circumference and BMI, however participants in the intervention group were significantly younger, had made more previous attempts to reduce weight and were more often employed full-time (Table 1).

**Table 1:** **Baseline characteristics with enrolment (complete**-**case analysis)**

	**Intervention**			**Usual care**				
	**n (%)**	**Mean**	**sd**	**n (%)**	**Mean**	**sd**	**Δ mean**	**p**
**Age (years)**	76	46,5	10,9	72	50,9	15,3	4,4	**0**,**045**^a^
**Weight (kg)**	76	96,4	21,5	72	95,4	20,2	1,0	0,759^a^
**Waist circumference (cm)**	73	110,9	17,6	60	107,3	13,7	3,6	0,194^a^
**Height (cm)**	76	169,3	0,1	72	168,7	0,1	0,6	0,703^a^
**BMI (kg**/**m**^**2**^**)**	76	33,6	7,0	72	33,3	5,3	0,3	0,791^a^
**Diets**	76	5,5	9,0	71	2,9	4,3	2,6	**0**,**011**^b^
**Gender**								0,388^c^
Females	53 (69,7)			45 (62,5)				
Males	23 (30,3)			27 (37,5)				
**Employment status**:		1,3	0,8	1,0		0,3	0,3	**0**,**030 **^b^
Full time	39 (52,0)			26 (36,1)				
Part time	22 (29,3)			23 (31,9)				
Not employed	14 (18,7)			23 (31,9)				

p-values are from ^a^Student t-Test, ^b^Mann-Whitney U-Test, ^c^Chi squared / Fishers’s exact Test (*sd *standard deviation, *Δ_mean *mean difference).

On the average, participants in the intervention group completed 6.4 (SD 4.2) week modules of the web-based coaching program. The mean duration of the program use was 72.7 (SD 28.7) days. This means that some participants did not use the program continuously and interrupted the program use (e.g. because of vacation). Weight decreased by 4.2 kg in the intervention group and by 1.7 kg in the control group. The reduction of waist circumference and decrease of BMI was also significantly stronger in the intervention group (see Table 2). The group differences for weight [1.3 kg (95%CI: 0.1; 2.5; p < 0.028)], waist circumference [2.6 cm (95%CI 1.0; 4.1; p < 0.001)] and for BMI 0.5 kg/m^2^ [(95%CI 0.05; 0.9; p = 0.029)] remained significant in the intent-to-treat analysis (data not in table). The secondary analysis using generalized estimating equation showed also significant differences for weight [(3.0 kg (95%CI 1,5; 5,5; p = 0.001)], waist circumference [4.6 cm (95%CI 2.8; 7.7; p < 0.001)] and for BMI [(1.0 kg/m^2^ (95%CI 0.5; 1.8; p < 0.001)] (data not in table). Self-reported eating behaviour and physical activity improved significantly more in the intervention group than in the control group while there were no significant differences regarding conscious eating and frequency of cooking (Table 3).

**Table 2:** **Results of weight loss**, **waist circumference and BMI regarding to the complete case analysis**

	**Complete-case analysis**							
	**Intervention**			**Usual care**			**Difference between conditions**	
	**n**	**Mean**	**sd**	**n**	**Mean**	**sd**	**Mean (95%CI)**	**p**
**Weight (kg)**								
Baseline	76	96,4	21,5	72	95,4	20,2	1, 0 (-5,7; 8,1)	0,759
Outcome	76	92,2	20,5	72	93,7	20,5	- 1,5 (-8,1; 5,2)	0,672
Difference	76	4,2	4,3	72	1,7	4,1	2,5 (1,1; 3,8)	<**0**,**001**
**Waist circumference (cm)**								
Baseline	73	110,9	17,6	60	107,3	13,7	3,6 (-1,9; 9,1)	0,194
Outcome	71	104,4	15,5	64	106,0	14,1	- 1,6 (-6,6; 3,5)	0,536
Difference	68	6,9	6,9	56	2,4	5,0	4,5 (2,3; 6,7)	<**0**,**001**
**BMI (kg**/**m**²**)**								
Baseline	76	33,6	7,0	72	33,3	5,3	0,3 (-1,7; 2,3)	0,791
Outcome	76	32,1	6,7	72	32,7	5,5	- 0,6 (-2,6; 1,4)	0,568
Difference	76	1,5	1,4	72	0,6	1,4	0,9 (0,4; 1,3)	<**0**,**001**

p-values are from Student t-Test.

**Table 3:** Results of eating behaviour and physical activity (complete case analysis)

	**Intervention**			**Usual care**				
	**n**	**Mean**	**sd**	**n**	**Mean**	**sd**	**Δ mean**	**p**
**Eating behaviour**:								
Baseline	76	3,2	1,0	72	3,3	0,9	0,1	0,918
Outcome	75	4,0	0,9	72	3,6	0,8	0,4	**0**,**006**
Difference	75	-0,8	1,1	72	-0,35	1,0	0,4	**0**,**008**
**Conscious eating**:								
Baseline	75	1,2	0,7	72	1,1	0,7	0,1	0,174
Outcome	75	1,8	0,4	72	1,5	0,7	0,3	**0**,**012**
Difference	74	-0,5	0,7	72	-0,4	0,7	0,1	0,509
**Frequent cooking** / **cooked meals**:								
Baseline	76	1,6	0,7	72	1,5	0,6	0,1	0,478
Outcome	75	1,7	0,6	71	1,5	0,6	0,2	0,155
Difference	75	-0,1	0,6	71	0,0	0,4	0,1	0,276
**Physical activity**:								
Baseline	75	1,5	1,2	72	1,2	1,3	0,3	0,071
Outcome	74	2,2	1,2	72	1,5	1,4	0,7	**0**,**003**
Difference	73	-0,6	1,2	72	-0,3	1,0	0,3	**0**,**048**

p-values are from Mann–Whitney U-Test.

## Discussion

We found that the web-based coaching program in combination with an accompanied telephone counselling and monitoring in general practice was effective to reduce weight in obese patients. Participants in the intervention group showed a weight reduction of 4.2 kg whereas participants in the usual care group reduced their weight only by 1.7 kg.

Some trials have already shown that weight loss programs could be effectively delivered via the Internet [14-17]. All of these successful online programs included a structured approach to modify energy balance and using cognitive-behavioural strategies such as self-monitoring. Our findings contribute to the growing body of evidence supporting the usefulness of the Internet as a platform for delivery weight loss interventions. Especially enhanced web-based interventions, which are tailored to the individual, showed higher effect sizes than solely informative websites [14]. However, this is the first study which examined a web-based intervention coordinated in general practice without the personal presence of patients during the weight reduction process. Bennett et al. [18] demonstrated with a randomized controlled trial in primary care that the participation of a 3 month web-based behavioural intervention was associated with 3.05 kg greater weight loss than usual care alone. This result is well comparable to ours (2.5 kg) even though the intervention of Bennett et al.’s study was accompanied by a coach support of registered dieticians which conducted at least two in-person and two telephonic counselling sessions. Another randomized controlled trial by Rothert et al. [14] received a mean group difference of weight loss from 1.4 kg after a web-based intervention of 3 months. This individually tailored weight management intervention was conducted without any coach support which might explain the lower efficacy when compared to our trial.

Our findings were similar to Tate et al. [19], who discovered a weight loss of 4.1 kg after 3 months within their intervention. They used a comprehensive website which provided a supply of food and physical activity diaries which was combined with regular (up to 5 times a week) contacts from a human counsellor via e-mail. The amount of weight loss in this study increased up to 5.3 kg after continuing the intervention for 12 months. A more recent study from Tate et al. [15] resulted in 5.3 kg of weight loss after 3 months by using an interactive website with a computer-automated feedback. However, they recommended additionally meal replacements consisting of specific liquid weight loss beverages.

Our significant result of weight loss within the intervention group was accompanied by simultaneous decrease of the waist circumference. The decreased waist circumference of 6.9 cm is similar to previously described findings [19,20]. Moreover, the mean decrease of the BMI by 1.5 kg/m^2^ is comparable to previous findings [18,19]. The results of the self estimated eating behaviour and physical activity of participants after intervention also revealed a significant improvement when compared to usual care patients and therefore confirmed previous findings [20] as well.

Our result, however, was somewhat lower than comparable trials of similar length with the presence of an interpersonal coach support. For example the result of a trial from Polzien et al. [21] showed a weight loss of 6.2 kg through 7 in-person individualized counselling sessions beside a web-based monitoring of energy intake and the use of a daily wearable body monitor.

When comparing commercial programs with an interpersonal coach support and general practice care including face-to-face setting [8], the results showed that the highest weight loss after 3 months with 4.4 kg had been achieved by participants in the Weight Watchers program. This finding is almost equal to our present result; our intervention, however, is only based on web and telephone coaching support and does not require a participant’s actual presence.

The great advantage of a structured web-based intervention is that the continuous personal presence of all involved parties is no longer necessary. Further advantages are the unlimited availability and free scheduling of a web-based weight reduction program. Some obese patients, for example, perceive face-to-face settings within a structured weight loss program as a burden [21] and weekly in-site visits are more time-demanding and cost-intensive than modern web based interventions [22,23]. The constant and widespread availability of a web based intervention program takes the increasing breakthrough of Internet use nowadays into account. Our trial demonstrated that a web-based weight loss intervention is generally feasible in primary care and to some extent also comparable to the known efficacy of several commercial interventions; its structure is more flexible, less time- and cost-consuming, reaches a broader population and has lower implementation costs.

### Strengths and limitations of the study

Strengths of the present study were the embedding of the study in a realistic primary care setting and the high consistency of the study results within different outcome measures. However, some important methodological aspects for the interpretation of the study results need to be considered. First of all, the randomization of the present study was conducted on practice level before individual participants were included. Thus, physicians knew whether they recruited patients for the intervention or the control group which could lead to bias. Baseline differences between intervention and usual care concerning the group size and some characteristics suggest that the recruitment was not conducted identically. The baseline difference in age could lead to the assumption that younger patients had higher internet skills so that our general findings possibly do not apply to older patients. The difference in patients with diet experience could also lead to the conclusion that patients with a larger history of diets had more motivation. The difference in employment status might be based on the fact that participants in the intervention group were younger. Including potential confounder variables into the covariance analysis tries to take this risk into account; it revealed that the observed group differences could at least not be explained with these confounders.

Secondly, due to the highly variable cluster sizes the statistical analysis of our data was not straightforward. Classical linear mixed models taking the cluster design into account could not be used because of numerical problems. Therefore, and because of the high number of practices with only one patient, we used simple t-tests (which ignores intra-cluster correlation) and an additional multilevel analysis (which runs with problems when cluster sizes differ) as sensitivity analysis. The results of both approaches are very similar giving credibility to our findings.

Thirdly, the proportion of participants without 12-week values was undoubtedly higher in the intervention than in the usual care group. This could be partly due to the fact that participants in the control group received a small financial incentive while those in the intervention group did not. Participants in the intervention group might also have been less willing to have an additional practice visit after completing the program than those in the control group who had little practice contact otherwise. Therefore, our complete case analysis might overestimate the difference between the groups to some extent. Within the intent-to-treat analysis, where the missing post values were replaced with a difference of 0 (baseline carried forward), the group differences appeared clearly smaller but remained statistically significant.

## Conclusion

In conclusion, our findings suggest that the tested web-based coaching program in combination with an accompanied telephone counselling and monitoring can effectively contribute to weight reduction in general practice on a short term basis. The effectiveness is comparable to other commercial interventions which are based on personal or group counselling sessions. Further RCTs are desirable in order to confirm present findings in larger populations and to investigate long-term outcomes.

## Competing interests

This study was completely funded by HausMed eHealth Services GmbH (Berlin, Germany). The sponsor did not have access to study data and did not influence the development of this manuscript. AS, KL, MM, SW are employed at University Hospital Klinikum rechts der Isar, Technische Universität München. JB and FF are employed at the HausMed Services GmbH. MH is medical student. There were no other financial and non-financial competing interests.

## Authors’ contributions

AS, KL and MM designed the study. MM wrote the initial protocol with supervision from AS and KL. MM coordinated with MH, FF and JB the study. MM, KL, AS, MH and SW did the analysis. MM drafted the manuscript with contribution from AS, KL, SW. All authors read and approved the final manuscript. MM is the guarantor.

## Authors’ information

Dr. med. Michael Mehring is in training as a general practitioner. Since February 2011 he has been active as a research assistant at the Institute of General Practice, Technische Universität München, Munich, Germany.

## Pre-publication history

The pre-publication history for this paper can be accessed here:

http://www.biomedcentral.com/1471-2296/14/76/prepub
